# The role of non-governmental organizations in strengthening healthcare systems in low- and middle-income countries: Lessons from Santé Diabète in Mali

**DOI:** 10.1080/16549716.2022.2061239

**Published:** 2022-05-09

**Authors:** Stéphane Besançon, Assa Sidibé, Djeneba Sylla Sow, Ousmane Sy, Julien Ambard, John S. Yudkin, David Beran

**Affiliations:** a NGO Santé Diabète, Bamako, Mali; b Hôpital du Mali, Bamako, Mali; c Ministry of Health, Bamako, Mali; d Independent Epidemiologist, Paris, France; e Division of Medicine, University College London, London, UK; f Division of Tropical and Humanitarian Medicine, University of Geneva and Geneva University Hospitals, Geneva, Switzerland

**Keywords:** Diabetes, nongovernmental organisations, health systems, NGO

## Abstract

Non-governmental organizations play a vital part in the achievement of the Sustainable Development Goals as defined by the United Nations. These Goals also include targets related to noncommunicable diseases. However, non-governmental organizations have played a limited role in this area despite such diseases causing the bulk of morbidity and mortality worldwide. Through their activities, non-governmental organizations should aim to strengthen health systems, yet they often only support these for a single disease. Mali, like many other low- and middle-income countries, is facing an increasing burden of diabetes and a health system not adapted to address this challenge. Santé Diabète, a non-governmental organization based in Mali since 2003, has been working specifically on diabetes, and has developed a wide range of activities to improve the national health system. This paper describes changes in the diabetes environment in Mali between 2004 and 2018 based on two health system assessments carried out using a Rapid Assessment Protocol. Over this period, the health system was strengthened with regard to financing and access to medical products. Leadership and governance, service delivery and health workforce were all improved but still partially rely on sustained support from Santé Diabète. The key lesson from this study is that to be effective in changing the management of noncommunicable diseases in a low- and middle-income country, non-governmental organizations need to play a variety of roles, many of which may change over time.

## Background

Non-governmental organizations (NGOs) play a vital part in the attainment of the United Nations. Sustainable Development Goals (SDG) to: ‘raise awareness and mobilize; build capacity; design and implement projects; monitor and review policies; collect data; provide technical expertise, and both support and hold governments accountable to their commitments.’[1] In the area of noncommunicable diseases (NCDs), NGOs have played a limited role despite NCDs representing the largest burden of morbidity and mortality worldwide [2,3].

The World Health Organization (WHO) prioritizes five NCDs: cardiovascular diseases, diabetes, cancers, chronic respiratory diseases and mental health [4]. In response to this global challenge, the WHO has developed a Global Action Plan (GAP) and established a High-Level Commission on NCDs. The GAP and High-Level Commission Reports call for a strengthening of health system responses to NCDs and emphasize the link between NCDs and Universal Health Coverage (UHC) [4–6]. These documents also highlight that NGOs should be involved in this global response, without detailing the specific roles [4–6]. The focus in these documents is more on NGOs as partners than as actors for change [6].

The roles of NGOs in global health and strengthening health systems include: working in partnership with Ministries of Health (MoH); managing joint projects and programs; piloting innovative service delivery approaches; involving communities; developing human resources, and strengthening infrastructure and information systems [7–9]. However, in some cases, this assistance has been ‘vertical’, focusing on a specific disease (e.g. HIV/AIDS), and not seen as strengthening the overall health system [10].

Chee et al. [11] distinguish between health system support and health system strengthening. Health system strengthening requires making comprehensive changes that durably impact the six health system building blocks proposed by WHO [12]. In contrast, health system support includes activities that improve how the health system functions by providing additional inputs that have a narrow or short-term focus [11].

Mali is located in West Africa with a population of about 20 million, a life expectancy at birth of 59 years and a Gross National Income per capita of US$ 840 [13]. The country is ranked 184 out of 189 countries on the Human Development Index [14] and faces a humanitarian crisis due to the ongoing military conflict [15]. The current 10-year health system plan (2014–2023) includes objectives such as: maternal and child health; communicable diseases; NCDs; the environment; health emergencies; health system strengthening, and governance [16]. The NCD component in this plan comprises activities in the areas of prevention, management, research, strengthening partnerships and coordination, and surveillance. The Malian health system is structured around Community Health Centers (Centres de Santé Communautaire, or CSCOMs), which are private not-for-profit associations managed by communities. CSCOMs are staffed by a doctor, a midwife and a nurse, who provide general medical consultations, pre- and postnatal consultations and assist with simple deliveries [17]. They also play a role in linking with communities. Only 58% of the population lives less than 5 km from a facility providing these basic services [17]. One of the reasons for this is the lack of health professionals and their unequal distribution within Mali [18]. In addition, households in Mali face high out-of-pocket expenditures for healthcare [18]. For some health issues, (e.g. HIV/AIDS, tuberculosis, caesarian deliveries, malaria testing for pregnant women and children under five, as well as surgery for fistulae) different programs provide care for free, funded by donors or the government [19].

Since 2009 the government has initiated Obligatory Medical Assistance (AMO), which is a health insurance scheme financed by employee and employer contributions in both the public and private sectors [20]. The AMO covers 80% of hospital and 70% of ambulatory care expenses. The government has also created the Medical Assistance Regimen (RAMED), which offers free services for the most vulnerable in Mali (about 5% of the population are considered as such) [20]. A system of Voluntary Medical Insurance (AMV) for people working in the informal sector is also available through community health insurance schemes. In June 2018, the Malian government legislated a Universal Health Insurance Regimen (RAMU) which aims for UHC [20].

## Santé diabète: a diabetes NGO in Mali

The International Diabetes Federation estimates that about 152,000 adults in Mali (aged 20–79) live with type 2 diabetes (a 2.1% prevalence) [21] and another 1,550 people with type 1 diabetes [22]. Santé Diabète (SD) is a French NGO based since 2003 in Mali, working specifically on diabetes throughout the country. The NGO started its work in Mali because several founding members had carried out research work on fonio (a West African millet/cereal) and diabetes in Mali, forging a close collaboration with medical specialists in the country. From this, a variety of challenges were identified which resulted in the implementation of the first Rapid Assessment in 2004, which was aimed at documenting these problems more clearly.

SD has positioned itself as both providing technical assistance to the MoH as well as implementing activities agreed upon by the MoH and other partners. These activities include: a peer education program for type 2 diabetes; education ‘days’ for children with type 1 diabetes; diabetes training for health professionals at national hospitals; training of diabetes referral doctors at regional hospitals and referral health centers (CSREF); a one-year and four-year University training program in endocrinology, metabolic diseases and nutrition at the Faculty of Medicine and Odonto-stomatology of Mali; a working group created by the National Health Directorate and SD to improve the supply and availability of diabetes medicines in Mali, as well as to negotiate prices of medicines; ensuring availability of diagnostic equipment adapted to each level of the health system; increasing awareness of diabetes in the population through education campaigns (communities and schools) as well as in the media, and the development and roll-out of patient education material for healthcare providers. Many of these initiatives in other settings would be the responsibility of actors such as the government, academia, diabetes associations or professional medical associations. All these activities were funded by SD from different sources, including development grants from governments and private foundations, support from regional authorities in France and research funding for specific initiatives.

The aim of this study was to investigate how an NGO can strengthen health services for diabetes care in sub-Saharan Africa. We used WHO’s health system building blocks [12] as a framework for analysis, linking these to the different roles NGOs can play [1].

## Methods

This study was based on data from two health system assessments in Mali that were carried out in 2004 and 2018. Both studies used the Rapid Assessment Protocol for Insulin Access (RAPIA) [23], which has been used in different low-and middle-income countries (LMICs) to assess access to insulin and diabetes care [24–27]. The protocol includes a multi-level assessment using interviews with semi-structured questionnaires, site visits, secondary analysis of existing data and document reviews (Table 1). Because the information collected is largely qualitative and semi-quantitative, no formal statistical comparison between the RAPIAs in 2004 and 2018 was performed. Any quantitative data are presented using descriptive statistics. All prices are standardized to 2018 US$ [28,29].

**Table 1.** Level and number of interviews carried out in 2018 and total interviews in 2004

	2018									Total 2004
	Bamako	Gao	Kayes	Koulikoro	Mopti	Segou	Sikasso	Timbuktu	Total	
Level: Macro (Ministry of Health, Central Medical Stores, NGOs, Civil Society, Ministry of Finance, Private sector)	*10*								10	20
Level: Meso (Regional Health Authorities, Regional Medical Stores, health facilities, private clinics, laboratories, pharmacies)	*25*	*6*	*6*	*9*	*6*	*11*	*15*	*5*	83	47
Level: Micro (health professionals, traditional healers and people with diabetes)	*94*	*19*	*18*	*28*	*29*	*29*	*45*	*16*	269	83
Total	*129*	*25*	*24*	*37*	*26*	*40*	*60*	*21*	362	150

The protocol was approved by the Comité d’Éthique de la Faculté de Médecine et d’Odonto-Stomatologie, Bamako, Mali and informed consent was sought and obtained from all participants. Data was collected by trained field workers. All questionnaires were in French. Translation to local languages was done when necessary, mainly for interviews with people with diabetes. Interviews were carried out with different individuals at various levels of the health system (Table 1). All interviewees were selected purposively. Interviewees responding at the macro-level were individuals from different government ministries and organizations, diabetes associations as well as clinical or academic opinion leaders. At the meso-level questionnaires targeted individuals from regional health authorities, health facilities, public and private laboratories, or public and private pharmacies. Finally, at the micro-level respondents were health professionals, traditional healers and people with diabetes. All interviews used adapted questionnaires targeted for the different interviewees covering the following themes: national policies and programs on NCDs and diabetes; policies impacting the importation, pricing and distribution of medicines and health-related supplies; organization of diabetes care; pricing and distribution of insulin, diabetes medicines and other related supplies; treatment and management of people with diabetes; barriers to care, and costs related to diabetes [23].

All data (interviews, document reviews, etc.) from the RAPIA in 2018 were entered in an Excel spreadsheet and analyzed using thematic analysis across different interviews and sources of information using the six health system building blocks proposed by the WHO as a framework [30]. These results were then compared with the data from the RAPIA in 2004 [31]. The results were also presented linking the six health system building blocks with the different roles played by NGOs [1].

## Results

A total of 362 interviews were carried out in nine regions of Mali in 2018 (Table 1), compared to 150 interviews in three regions in 2004. The results are presented for each of the six WHO building blocks.

### Leadership and governance

Activities implemented by SD for this health system component focused on raising awareness of diabetes as a health problem in Mali, as well as designing and implementing projects in collaboration with the MoH. In addition, SD provided technical expertise to the MoH for the development of a new strategy on NCDs running from 2019 to 2023. SD also ensured that NCDs and diabetes were included in the current health system plan (2014–2023) [16]. Since 2004, this work has been facilitated by SD through a memorandum of collaboration with the MoH. In addition, an ad-hoc group bringing together different governmental and non-governmental partners was established, to ensure synergies and to create space for dialogue around different issues regarding the prevention and management of diabetes. More recently, this group was restructured as a steering committee, created by Ministerial decree, that includes patient organizations, professional medical associations, communities, other governmental organizations and SD.

In 2004, a draft NCD strategy was in the process of being developed. In 2018, the national NCD strategic plan (2015–2019) [32] covered very specific activities with regard to diabetes, including: screening for diabetes, linked with health promotion and sensitization activities for the population and health professionals; provision of insulin, oral medicines and other diabetes supplies (blood glucose meters, test strips and syringes) to patients; development of surveillance tools; defining referral pathways, and strengthening partnerships between different actors.

The health system plan established in 2014 included NCDs with specific objectives relative to NCD morbidity, mortality and disability linked to NCDs [16]. This strategy focused on certain key actions including improving the availability and affordability of medicines and other supplies, training of health professionals in the prevention and management of NCDs, integration of NCDs within basic training, and the creation of specialized units for NCD management at CSREF and hospitals. In 2018, SD also worked with the Malian government to ensure that gestational diabetes and care for pregnant women with diabetes was included in the National Maternal and Child Health Strategy.

In addition to these national guiding documents, each region visited in 2018 had an NCD focal point at the Regional Health Directorate. The role of these focal points was the regional implementation of the national strategy. At a national level, a diabetes-specific coordinating mechanism was created bringing together the MoH, SD, civil society, healthcare providers, Central Medical Stores and the National Agency for Telemedicine and Medical Informatics. This contrasts with 2004, when only one person was responsible for the national NCD and diabetes response.

### Service delivery

To address the challenges of service delivery, capacity building was an absolute necessity given how diabetes care was provided in 2004. Through designing and implementing different projects, as well as providing technical expertise, SD was able to strengthen diabetes care as well as decentralize services. Beyond direct health system activities, SD also implemented different activities relating to the education of people with diabetes, and a study on peer-led education [33]. This study and others carried out by the NGO also highlighted that the role SD played in data collection within the service delivery component had a role in increasing awareness, mobilizing local stakeholders, as well as informing further interventions.

Diabetes care in 2004 was mainly delivered in three places: two tertiary hospitals in Bamako, and a clinic run by the diabetes association. Outside of Bamako there were no referral hospitals with dedicated diabetes services, and care was dependent on the level of training of health professionals. In 2018 ‘l’Hôpital du Mali’ in Bamako has become the national referral center for diabetes with a specialized unit for children with type 1 diabetes. Today, 32 decentralized diabetes clinics are now operational throughout Mali, with each district hospital including a diabetes unit. Moreover, some CSREFs and CSCOMs in different regions and districts provided diabetes care. A problem with referrals was noted with individuals bypassing lower levels of the health system and going directly to the national referral center. Another persistent problem were the long waiting times in the facilities.

In the 2004 survey, many expressed feeling abandoned by the system and suffered from the lack of care outside the three facilities in Bamako. In 2018 most people with diabetes interviewed mentioned the need for increased therapeutic patient education as well as for other educational and informational tools. Fifty percent of facilities in 2018 had received educational support tools for people with type 2 diabetes via the work of SD and the MoH. Among interviewed health professionals, 92% stated they held education activities, however only 67% of people with diabetes stated having participated in such an activity. An evaluation of peer-led education activities in Mali found that these had a positive impact on Glycated Hemoglobin (HbA1c) [33].

The 2004 RAPIA highlighted the important role played by traditional healers in the overall delivery of care in Mali, but noted there was little interaction with allopathic medicine. Traditional healers’ roles and linkage with the health system remained the same in 2018.

### Health system financing

With regard to the financing of health care, SD has played a role in collecting data, providing technical expertise and advocating for the government to ensure that diabetes medicines and other aspects of care are included in the different insurance packages proposed by the Malian government.

Mali has seen dramatic changes in healthcare financing since 2004. Table 2 details the elements of diabetes care that were covered in 2018 by the different insurance schemes available to the Malian population as well as their strengths and weaknesses. Consultation fees in 2018 were FCFA 600 (US$ 1.08) in CSCOMs, FCFA 1,000 (US$ 1.80) in CSREFs and Regional Hospitals and FCFA 1,500 (US$ 2.70) for a specialized consultation at a national hospital. These fees allow for a medical consultation and the associated care received, and are included in AMO, AMV and RAMED baskets. In 2004, these fees varied from FCFA 200 (US$ 0.50) to FCFA 1,000 (US$ 2.52). In contrast, in 2004 most care was provided by the diabetes association clinic and people had to pay membership fees and consultation fees to receive their care, on top of the cost of medicines.

**Table 2.** Strengths and weaknesses of different insurance schemes in Mali relative to diabetes

Insurance scheme	Strengths	Weaknesses
**AMO**	Large number of public and private facilities included (health facilities and pharmacies) 70% coverage on insulin and oral medicines	Only for formal sector (less than 20% of Malian population) Blood glucose meters and strips not included
**AMV**	Large number of public and private facilities included (only pharmacies) 75% coverage of medicines included on Malian essential medicine list (Metformin, Glibenclamide and Insulin)	Lack of awareness of this scheme High cost of monthly premium (FCFA 575 (US$ 1.03)) Blood glucose meters and strips not included Other oral diabetes medicines are not included
**RAMED**	100% coverage of medicines included on Malian essential medicine list	Lack of awareness of scheme Assessment of need obscure and length bureaucratic process Blood glucose meters and strips not included Other oral diabetes medicines are not included

### Health workforce

One of the main weaknesses found in 2004 was a lack of trained health professionals. Since then, SD has focused on building capacity through different training programs as well as designing and implementing different projects. In addition, it coordinated technical support from a variety of external partners in the area of healthcare worker training. In 2018, 32 focal diabetes doctors collectively managed more than 20,000 people in the different regions of Mali. With a total of 850 health professionals having received diabetes training this has boosted the development of human resources specialized in diabetes, as well as sustained diabetes training in continuous professional development.

Among interviewed health professionals, 93% of doctors and 82% of nurses who worked in diabetes consultations stated having received specific training in diabetes. In addition, the two University diploma training programs in endocrinology, metabolic diseases and nutrition enabled a total of 59 students from Mali to receive advanced certification courses in diabetes and endocrinology. This allowed for the development of human resources specialized in diabetes as well as strengthening diabetes training in continuous professional development. This contrasts with a lack of training in diabetes in 2004 besides training in medical school and learning-on-the-job in one of the diabetes-specific consultations.

### Medical products and technologies

**Figure 1.**
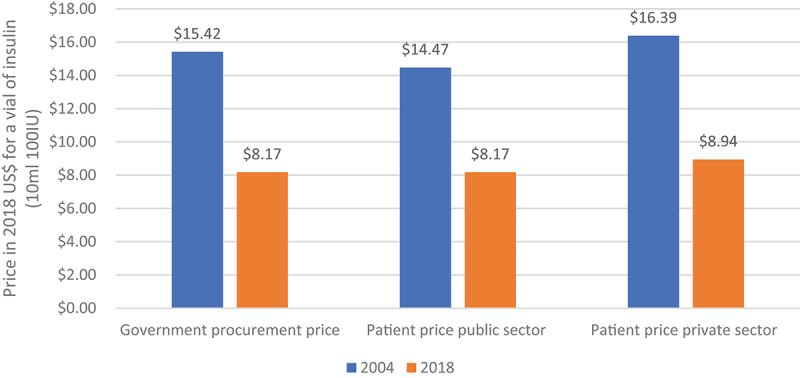
Government procurement and patient public and private prices for a vial (10 ml 100IU) insulin (2018 US$).

Another important change between 2004 and 2018 was the increased availability of insulin. In 2004 insulin was only available in 17% of facilities (public and private). In 2018 in contrast, 65% of facilities visited had at least one type of insulin. Prices in the public and private sectors also decreased, and became more uniform (Figure 1). Data on oral medicines was not collected in 2004, but data from 2018 showed a wide range of prices within and between the sectors. For example, Glibenclamide (5 mg) varied 26-fold in price in the private sector. Between public and private sectors, the average prices for Glibenclamide and Metformin (850 mg) were 3.4 and 2.6 times more expensive in the private sector, respectively. In 2004 syringes were only available in the private sector, at an average cost of US$ 0.43. In contrast in 2018, syringes were available in 44% of public and 93% of private pharmacies. The average price had halved, to US$ 0.22 and US$ 0.24 in public and private pharmacies, respectively.

Another positive change between 2004 and 2018 was the availability of diagnostic tools, including blood glucose meters, urine ketone strips and other analytics. In 2004, HbA1c tests were available only in one private sector laboratory at a cost of US$ 25.72, in contrast to US$ 14.75 and US$ 12.68 in 2018 in the private and public sectors, respectively (2018 prices). In 2018, 60% of national hospitals, 50% of regional hospitals and 56% of CSREFs had equipment and consumables to carry out this test.

These changes in the availability and affordability of medicines and related supplies were due to awareness raising and collaboration between the MoH and SD. For insulin access, SD provided technical support as well as advocacy. For HbA1c, SD together with the MoH organized supplies, with SD purchasing the analytical equipment and initial reagents and transferring these to facilities, followed by sustained supply by the MoH.

### Health information systems

In 2004, there had been a complete lack of standardized tools for data collection at facilities. Diabetes registries and individual patient records now exist thanks to SD projects, which required such data to monitor and evaluate the implementation of their projects. Ninety percent of facilities visited in 2018 had a consultation register created by the National Health Directorate and SD. A specific database for type 1 diabetes was developed. This diabetes data is not yet routinely collected centrally by the health system, but work is ongoing to integrate this into the District Health Information Software (DHIS2) in Mali.

### Other areas of activity

Three other areas of improvement are worth noting. Firstly, the creation of the Malian National Federation of Diabetics (FENADIM) with its local branches in all regions (30 in total). This is yet another change from 2004 when the association was only active in the capital, with some activities in Sikasso in the south of the country. SD provided capacity building for the FENADIM with regard to management, governance, and developing the role of the association in the Malian context. Secondly, different health promotion and prevention activities have been developed and implemented by SD. These include: World Diabetes Day events with screening campaigns; awareness raising, and school-based health promotion around nutrition and physical activity. Finally, SD has shown its capacity for innovation by supporting the humanitarian response in northern Mali in 2012 [34] and, since 2017, also working to integrate the services provided for diabetes with those for HIV/AIDS and tuberculosis in Mali. These other areas of activity cut across the different roles of an NGO resulting in general awareness raising, capacity building, design and implementation of projects, collection of data, and provision of technical expertise roles.

## Discussion

SD is playing a significant role in raising awareness and providing support to the Malian government, with health system financing and access to medical products being areas where the NGO has enabled the health system to be strengthened. Leadership and governance, service delivery and health workforce fall in-between health system support and its strengthening. Although progress was made, these areas are still dependent on SD input, and sustained funding and technical support are not yet fully integrated into the health system. For example, the health workforce component has clearly shown improvements between 2004 and 2018 in terms of the number of personnel trained, how these professionals work within the Malian health system and how specific training courses have been put into place. However, SD in many cases organizes and funds these trainings through external support and these are not yet part and parcel of the health system. The health information systems component is an example of health system support, in that not all the data collection tools used for diabetes are currently integrated within the Malian health system.

**Figure 2.**
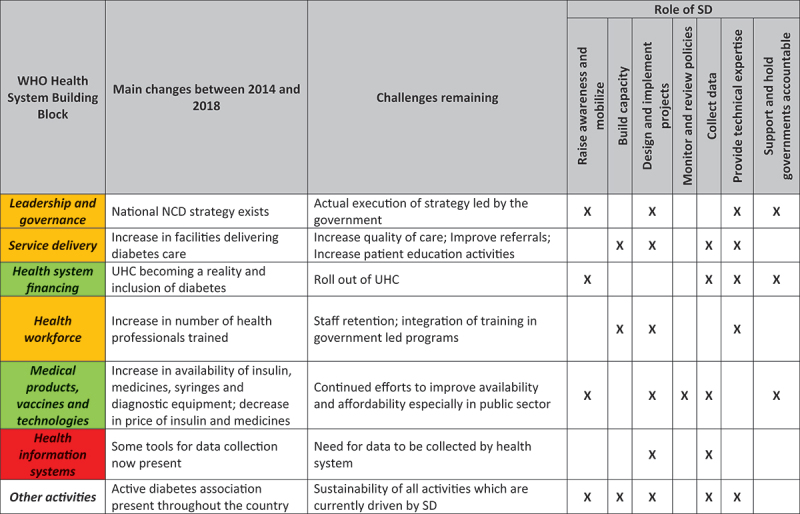
Summary of health system support or strengthening activities and role SD played in this.

The design and implementation of projects led by NGOs is a shared component that underpins all these activities (Figure 2). The experience with SD shows that an important first step was the initial assessment of the health system using the RAPIA in 2004; this was followed by targeted activities. The other lesson, in a context like Mali, is that in order to strengthen health systems for effective management of NCDs and diabetes, NGOs have to play a variety of roles, which may evolve over time.

Health system support (shown in red) versus strengthening (in green). Areas falling in-between health system support and health system strengthening (shown in orange)

This study was descriptive in nature rather than a formal evaluation, and it is impossible to quantify exactly the impact of SD’s work on the various areas of the health system. But it can be argued that SD’s work has been catalytic, and its impact can be indirectly measured by the increase in people diagnosed with type 1 diabetes. In 2004 only 14 people with type 1 diabetes were registered, in comparison to 584 in 2018 [31,35]. As argued previously, type 1 diabetes is a useful tracer condition for assessing health systems [24,36,37] and this increase in number of people with type 1 diabetes within the system can be seen as a proxy for improvements in the management of diabetes in Mali, and the impact of the work of SD.

An analysis in Mozambique found similar impacts of this approach to improving diabetes care between 2003 and 2008 [38] and in Rwanda health system support activities have shown improvements in the numbers of people with type 1 diabetes due to increased survival [39]. What is unique with the example of SD is how, beyond its direct impact on health system strengthening that the approaches developed by SD have had, this NGO has played a role in addressing diabetes for the Malian population in an LMIC by fulfilling the roles of other actors. Society can be portrayed as a three-legged stool with one leg being the public sector that protects the health and well-being of its population; a responsible private sector that supplies the products needed, and finally civil society that provides the links between communities [40]. In the Malian context, SD, which is a part of the ‘civil society’ leg, fulfils roles of both public and private sectors within the complex context of diabetes.

The limitations of this study are that, although it compares 2004 and 2018, data was not collected at the same facilities, and is descriptive in nature with no specific outcomes being assessed. Overall, the sample is purposive and is relatively small. In addition, the results present two snapshots rather than a true evolution of the situation over time. As with all mixed method approaches with interviews and observations, there are both interviewer and interviewee biases. Another source of bias in the analysis of the data is that three of the authors (SB, AS and DB) are intimately linked to the work of SD.

NGOs or civil society in diabetes and NCDs in general have to a large extent been absent or focused primarily on advocacy and awareness-raising rather than actual implementation of programs in a given country. In sub-Saharan Africa, the focus for diabetes associations has been on advocacy and provision of care if this was lacking [41]. The example of SD highlights that, to address the global NCD burden, local NGOs should be empowered to be a force for change in addressing this challenge in their local contexts.

## Paper context

NGOs are part and parcel of the global health landscape and play an important role in addressing health-related issues globally and nationally through various means. We present the experience from Mali where Santé Diabète, an NGO based in Mali since 2003, has implemented a variety of projects and programs. The lessons from this experience are that to be effective in changing the management of diabetes in a context like Mali, NGOs have to play a variety of roles and these may evolve with time. This example suggests that, to address the global diabetes burden, local NGOs should be empowered to be a force for change in addressing this challenge in their local contexts.
